# Efficacy and safety of rivaroxaban plus clopidogrel versus aspirin plus clopidogrel in patients with coronary atherosclerotic heart disease and gastrointestinal disease undergoing percutaneous coronary intervention: study protocol for a non-inferiority randomized controlled trial

**DOI:** 10.1186/s13063-023-07236-w

**Published:** 2023-03-21

**Authors:** Tienan Zhou, Yinghui Gong, Jingyuan Li, Yasong Wang, Xiaozeng Wang

**Affiliations:** Department of Cardiology, General Hospital of Northern Theater Command, No. 83 Wenhua Rd, Shenyang, 110016 Liaoning China

**Keywords:** Percutaneous coronary intervention, Gastrointestinal disease, Dual antiplatelet therapy, Rivaroxaban, Protocol

## Abstract

**Background:**

Dual antiplatelet therapy (DAPT) with aspirin and a P2Y_12_ inhibitor is recommended for patients with coronary heart disease (CHD) undergoing percutaneous coronary intervention (PCI) to antithrombosis, meanwhile, increasing the risks of gastrointestinal bleeding. Rivaroxaban, a novel oral anticoagulant, combined with a P2Y_12_ receptor inhibitor reduces adverse events in patients with CHD and atrial fibrillation who underwent PCI. The effect of rivaroxaban plus P2Y_12_ inhibitor on reducing bleeding events in patients with CHD and gastrointestinal disease (GID) undergoing PCI remains unclear.

**Method:**

The study is a prospective, single-center, randomized controlled trial. A total of 1020 patients with CHD and GID undergoing PCI will be enrolled. Patients are randomized (1:1) to receive either rivaroxaban 10 mg plus clopidogrel 75 mg daily or aspirin 100 mg plus clopidogrel 75 mg daily; both treatments will last 6 months. The primary endpoint is Bleeding Academic Research Consortium (BARC) type 2–5 bleeding requiring medical intervention. The secondary endpoint is a composite of major adverse cardiovascular and cerebrovascular events (MACCE), including all-cause death, cardiac death, nonfatal myocardial infarction, stent thrombosis, ischemia-driven target vessel revascularization, and stroke.

**Discussion:**

The objective of this study is to evaluate the efficacy and safety of rivaroxaban plus clopidogrel versus aspirin plus clopidogrel in patients with CHD and GID undergoing PCI. We aim to explore an optimized antithrombotic strategy, which achieves the same anti-ischemic effect as standard DAPT without increasing the risk of GIB, for patients with CHD and GID undergoing PCI.

**Trial registration:**

This protocol is registered at the Chinese Clinical Trial Registry under the number ChiCTR2100044319. And this publication is based on version 1.4 of the trial protocol dated Sep 6, 2021.

**Supplementary Information:**

The online version contains supplementary material available at 10.1186/s13063-023-07236-w.

## Highlights

1. To find a more appropriate antithrombotic strategy for patients with CHD and GID undergoing PCI.

2. To identify whether rivaroxaban can reduce the bleeding risk of the gastrointestinal tract compared with aspirin.

**Supplementary Information:**

The online version contains supplementary material available at 10.1186/s13063-023-07236-w.

## Introduction

Antithrombotic treatment must be performed in patients with coronary atherosclerotic heart disease (CHD) undergoing percutaneous coronary intervention (PCI). Dual antiplatelet therapy (DAPT) with aspirin and P2Y_12_ inhibitor, which can reduce the risk of ischemic or atherosclerotic thrombotic events (including in-stent thrombosis, recurrent myocardial infarction, and cardiac death) is considerable [1, 2]. Both ischemia and bleeding events significantly influence the outcome of CHD patients [3]. Although DAPT can reduce the risk of thromboembolic events, it is also associated with a higher risk of major bleeding, the most common of which is a gastrointestinal mucosal injury such as ulceration and bleeding [4, 5].

For patients with concomitant *H. pylori* infection, antithrombotic therapy substantially increases the risk of upper gastrointestinal bleeding (UGIB) by 1.8 times (95% CI 1.5–2.1) with aspirin alone and by 7.4 times (95% CI 3.5–15) with DAPT [6]. And UGIB in patients with acute coronary syndrome (ACS) is associated with markedly increased mortality [7]. Previous gastrointestinal diseases (GID) and the combined anti-platelet therapy are the main risk factors for UGIB. Long-term treatment of aspirin inhibits the activity of gastrointestinal cyclooxygenase and results in epithelial to the gastrointestinal mucosa damage in patients with CHD and GID, increasing the risk of bleeding [8]. UGIB can lead to increased mortality and discontinuation of DAPT and failed adequate secondary prevention of CHD subsequently. Out of concern for gastrointestinal damage from DAPT, patients with CHD and GID are less likely to receive revascularization, increasing the risk of cardiovascular death in these patients.

The WOEST trial enrolled patients post-PCI who received oral anticoagulation using a different strategy by antithrombotic regimen, free of aspirin showed a significantly lower incidence of major bleeding among patients assigned to clopidogrel plus a vitamin K antagonist (VKA) (at therapeutic doses) than those assigned to triple antithrombotic therapy with aspirin plus clopidogrel plus VKA (at therapeutic doses) [9]. The PIONEER AF-PCI trial randomly allocated patients to three groups: rivaroxaban 15 mg once daily plus a P2Y_12_ inhibitor for 12 months; rivaroxaban 2.5 mg twice daily plus DAPT for 1, 6, or 12 months; or a dose-adjusted oral VKA plus DAPT for 1, 6, or 12 months. In the two groups receiving rivaroxaban, the rates of clinically significant bleeding were lower, but there were no significant differences between the groups in terms of stroke, myocardial infarction, or death from cardiovascular causes [10]. However, the population in this study was about 94% whites and only about 4% Asians. Previous studies have suggested that the bleeding risk of antithrombotic therapy seems to be higher in the Asian population than that in the white population. The Gemini-ACS-1 study demonstrated that low-dose rivaroxaban did not increase clinically significant bleeding when used with clopidogrel or ticagrelor compared to DAPT. Free of aspirin strategy may be a safe approach for ACS. No risk of thrombotic events due to the discontinuation of aspirin was found [11]. Those studies demonstrated that NOAC (rivaroxaban) as an alternative to aspirin plus a P2Y_12_ inhibitor could achieve similar anti-ischemic effects.

Currently, no clinical studies and guidelines have shown that rivaroxaban can be used instead of aspirin in antithrombotic therapy for patients with CHD and GID. Therefore, we designed a prospective, non-inferiority, randomized controlled trial to assess the efficacy and safety of rivaroxaban plus clopidogrel versus aspirin plus clopidogrel in patients with CHD and GID undergoing PCI.

## Methods

### Study design

**Fig. 1:**
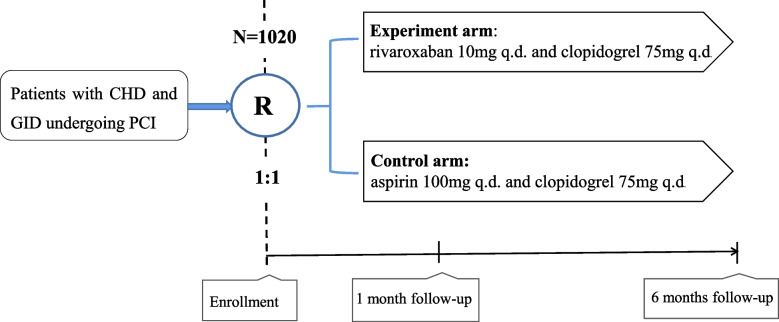
Flow chart of the study. CHD, coronary atherosclerotic heart disease; GID, gastrointestinal disease; PCI, percutaneous coronary intervention

The study is a prospective, single-center, non-inferiority, randomized controlled trial. A total of 1020 subjects will be recruited at the Section V of the Cardiology Department of the General Hospital of Northern Theater Command in China. To evaluate the efficacy and safety of rivaroxaban plus clopidogrel versus aspirin plus clopidogrel in patients with CHD and GID undergoing PCI. The study flow diagram is shown in Fig. 1. The SPIRIT Checklist is shown in Supplemental file 1 [12].

### Population

Eligible criteria are as follows: Patients 18 to 75 years of age with stable coronary atherosclerotic heart disease or non-ST segment elevation acute coronary syndromes (NSTE-ACS) combined with GID, Global Registry of Acute Coronary Events (GRACE) scores < 140 points, are considered to be enrolled. Patients with GIB or gastrointestinal ulcers that have healed for more than 12 months or the patients who take aspirin accompanied by uncomfortable but tolerable symptoms (e.g., stomachache, abdominal distension) are included. The diagnosis of gastrointestinal disease is determined by the physician based on the patient’s clinical symptoms, the result of the endoscopy, and previous medical history. GID is defined as acute or chronic gastritis, gastric mucosal erosion, GIB or gastrointestinal ulcer healing for 1–12 months, gastrointestinal dysfunction diagnosed by a specialist, or gastrointestinal tumor planning surgery. Patients who met any of the exclusion criteria are excluded. Main exclusion criteria include a GRACE score > 140 points in patients with non-ST segment elevation myocardial infarction (NSTEMI) or ST-segment elevation myocardial infarction (STEMI), severe renal insufficiency (creatinine clearance < 30 mL/min), and previous aspirin or rivaroxaban allergy.

Detailed inclusion and exclusion criteria are listed in Table 1. The subjects met all inclusion criteria and no exclusion criteria are eligible for this study after signing the informed consent form. The informed consent form is provided in Supplemental file 2. To ensure the safety of patients, we will continue to monitor and care for until the end of the study regardless of the reason why participants withdrew from the study. This trial will be terminated in case of any abundance of adverse events or intervention-related complications.

**Table 1:** Inclusion and exclusion criteria

**Inclusion criteria**
• 18 to 75 years of age
• Stable Coronary artery disease or NSTE-ACS with GRACE score < 140 points
• Gastrointestinal diseases
• Taking aspirin with a stomachache, abdominal distension, and other discomforts but can tolerate
• Informed consent
**Exclusion criteria**
• NSTE-ACS with GRACE score > 140 points or STEMI
• History of intracranial hemorrhage within one month or vital organ bleeding
• Platelet count < 100 × 10^9^/L
• Hemoglobin < 100 g/L
• Active hepatitis or ALT, AST values > 3 × the upper limit of normal
• Severe renal insufficiency (CrCl < 30 mL/min)
• Body weight < 45 kg
• History of aspirin or rivaroxaban allergy
• Not willing to undergo PCI
• Severe progressive disease (e.g., malignant tumor)
• Anticipated life expectancy < 6 months
• Pregnancy, breastfeeding, and childbearing plans
• Long-term use of (N)OAC
• Long-term use of strong CYP3A4 inhibitors or P-glycoprotein inhibitors
• Long-term use of moderately potent CYP2C19 inhibitors or CYP2C19 inducers
• Expected to be unable to tolerate medication for 6 months
• Unable to obtain an informed consent or the researchers think it is not suitable to participate in this trial in any case
• Participating in other ongoing clinical studies

*GRACE* Global Registry of Acute Coronary Events, *NSTE-ACS* non-ST-segment elevation acute coronary syndromes, *STEMI* ST-segment elevation acute myocardial infarction, *ALT* alanine aminotransferase, *AST* aspartate aminotransferase, *CrCl* creatinine clearance (CrCl = [(140-age) × weight (kg) × (0.85 female)]/[0.818 × Scr (mol/L)], *PCI* percutaneous coronary intervention, *NOAC* new oral anticoagulant

### Recruitment

Advertisement through posters and an outpatient publicity board is used to identify potential participants to reach the required sample size.

### Randomization and blind

Before randomization, all enrolled subjects are assessed for randomization. Subjects with any of the following conditions shall not be randomized: withdrawal of informed consent, voluntarily withdrawal from the trial, the investigator determining that random antithrombotic regimens cannot be accepted, and unwilling or unable to undergo PCI.

After the assessment, the subjects are getting a random number. Based on the number, subjects are randomized (1:1) to the experiment arm (rivaroxaban plus clopidogrel) or the control arm (aspirin plus clopidogrel) by the block randomization table. Two investigators enroll subjects. The random number was generated by an independent professional statistician using the SPSS 25.0 software. According to the order generates a random number and informs the number to the authorized doctor who obtains informed consent and decides the intervention. This trial is an open-label design. The investigators and the subjects know which drugs the subjects are using, but the independent event assessment committee and data analysts do not know which drugs the subjects are using.

### Interventions

Subjects assigned to the experiment arm should receive rivaroxaban 10 mg plus clopidogrel 75 mg daily. Subjects assigned to the control arm should receive aspirin 100 mg plus clopidogrel 75 mg daily. All subjects are given medication routinely for 6 months. If subjects had never or not routinely taken clopidogrel or aspirin in the pastor taken clopidogrel 75 mg or aspirin 100 mg daily routinely less than five days before inclusion, a loading dose of clopidogrel 300–600 mg or aspirin 300–600 mg should be given within 12 h before coronary angiography.

If GIB occurs during the study, antiplatelet drugs should be discontinued, endoscopic hemostasis under the condition of stable hemodynamics, and Proton Pump Inhibitors (PPI) drug treatment are performed. Three days after hemostasis, subjects are given clopidogrel 75 mg monotherapy daily as soon as possible if the gastric injury is localized or completely hemostatic, no active bleeding relapses, and hemoglobin remains above 90 g/L after blood transfusion. Five days after hemostasis, they return to the original treatment strategy after randomization. But if there is still a small range of active or occult bleeding and the hemoglobin fluctuates above 100 g/L on different days, subjects are given low molecular weight heparin. Seven days after hemostasis, they restore to the original treatment strategy after randomization, and low molecular weight heparin was discontinued at the same time.

### Schedule of assessments

After subjects provided informed consent and were enrolled in the study, the data collection is initiated. Investigators should collect information including demographic information, admitting examination, and laboratory tests.

At the follow-up visit of 30 days by telephone contact, investigators need to know about and record information as follows: (1) bleeding events, including location, time, treatment, hospitalization, and duration of hospitalization; (2) MACCE, including time of occurrence, treatment, hospitalization, and length of hospitalization; (3) reviewing concomitant medication with subjects; (4) informing subjects of the importance of compliance with study medication; (5) scheduling the next visit; (6) informing subjects and/or guardians to notify investigators of any adverse events.

At the follow-up visit of 6 months on-site, investigators should collect data as follows: (1) assessing MACCE; (2) assessing bleeding events, including location, time, treatment, hospitalization, and duration of hospitalization; (3) proceeding routine blood test and fecal occult blood test to observe whether the patient has gastrointestinal bleeding and other diseases; (4) reviewing concomitant medication with subjects. In terms of patient compliance with medication, the medication compliance of patients was judged by regular follow-up. At the follow-up visit of 30 days by telephone contact, investigators checked the amount of pills left by the subjects. During a 6-month on-site follow-up, pill boxes would be handed by subjects to check their medication compliance. At the end of the study, the physician decides whether subjects should be received which antithrombotic treatment and whether they should be received PPI and H2 receptor antagonists. Detailed schedule content is listed in Table 2. We will request consent for the review of participants’ medical records, and for the collection of blood samples to assess hematology (RBC, WBC, HGB, HCT, Plt) and fecal occult blood. Biological specimens collected from subjects will be used only for laboratory testing and will be destroyed safely when the test ends. No genetic or molecular analysis is scheduled.

**Table 2:** Study schedule

	Enrollment (Visit 1)	1 month ± 7 days (Visit 2)	6 months ± 7 days (Visit 3)
Inclusion/exclusion criteria	√		
Informed consent	√		
Demographic data	√		
Previous history	√		
Admitting diagnosis	√		
Admitting examination and laboratory tests	√		
Information of PCI	√		
Drug therapy	√	√	√
Hematology (RBC, WBC, HGB, HCT, Plt)	√		√
FOB	√		√
Bleeding event		√	√
MACCE		√	√

*PCI* percutaneous coronary intervention, *RBC* red blood cells, *WBC* white blood cells, *HGB* hemoglobin, *HCT* hematocrit, *Plt* platelets, *FOB* fecal occult blood, *MACCE* major adverse cardiovascular and cerebrovascular event

### Endpoints

The primary endpoint of the study is Bleeding Academic Research Consortium (BARC) type 2–5 bleeding events requiring medical intervention. The secondary endpoint is a composite of MACCE including all-cause death, cardiac death, nonfatal myocardial infarction, stent thrombosis, ischemia-driven target vessel revascularization, and stroke. Other endpoints include the separate components of the MACCE and BARC 3–5 bleeding. Detailed definitions of all study endpoints are provided in Supplemental file 3.

### Adverse event reporting and harms

Adverse events (AEs), serious adverse events (SAEs), and harms from interventions will be collected and reported to CEC. Those events include bleeding events (BARC) and other serious adverse events (e.g., death, ischemia-driven target vascular revascularization, stent thrombosis, and myocardial infarction). Furthermore, the investigator will be reported to relevant regulatory bodies as needed, indicating expectedness, seriousness, severity, and causality.

### Data management and confidentiality

Case Report Form (CRF) and entering the information into an electronic database will be completed by two investigators. They are double-checked by them each other. All the CRF on paper will be stored in a locked cabinet, using the phonetic initials of the name as the code name and access to the data is only available to those who are authorized by the principal investigator. All information collected in this study will be strictly protected to ensure the privacy of participants.

### Sample size

Based on the incidence of BARC type 2–5 bleeding in the GEMINI-ACS-1 [11] trial and OPT-CAD trial, provided that the incidence of BARC type 2–5 bleeding in the experiment arm and the control arm are both 6% at 6 months. A non-inferiority margin of 4.7% was estimated and based on a one-sided type 1 error of 2.5%, power of 85%, and a 10% drop-out rate, a total of 1020 patients was needed for adequate analysis. Sample-size determination was performed with SAS version 9.3.

### Statistical analysis

The analysis of the primary endpoint that BARC type 2–5 bleeding events require medical intervention for 6 months based on intention-to-treat (ITT) and per-protocol (PP) population. The ITT population is all randomized subjects. All randomized subjects without major protocol violations, subjects who did not receive the assigned treatment, or did not receive any treatment belong to the ITT population. The PP population is fully in line with the study. The study will conduct subgroup analyses of primary and secondary endpoints based on gender, age, and diabetes in the ITT population. Multiple imputation will be performed to handle missing data. The main endpoints of the ITT population are analyzed and then reanalyzed in the PP population to support the results. The analysis of the composite of MACCE is based on the ITT population. Unless otherwise stated, all hypothesis tests are carried out at a bilateral significance level of 5%. To determine whether the efficacy and safety of rivaroxaban plus clopidogrel in patients with CHD and GID are not inferior to aspirin plus clopidogrel. The difference in the incidence of endpoints between the experiment group and the control group is less than or equal to the non-inferior effect value means an invalid hypothesis (H0, P0–P1 ≤  − 4.7%). The difference in the incidence of the primary endpoint between the two groups is greater than the non-inferior effect value remains an alternative hypothesis (H1, P0–P1 >  − 4.7%). The non-inferiority test is carried out at a significance level of 0.025 on one side. Statistical analyses were performed with SAS version 9.3.

### Clinical Events Committee and Data and Safety Monitoring Board

The Clinical Events Committee (CEC) is composed of medical experts in the field of cardiology and gastroenterology. Committee members are responsible for reviewing all reported events and classifying those events. If necessary, CEC can request original information from the research center. Members are remaining blind to treatment assignments.

The study is monitored by an independent Data and Safety Monitoring Board (DSMB). All adverse events in this study will be reported to the DSMB, and they can request additional information as required. The DSMB will regularly check the endpoint events to identify the safety of the study. DSMB will supervise and manage the research scheme and data before the start of the study, mid-study, and after the end of the study. Based on the result of the review, the DSMB will provide a report about the safety of the study to the lead investigator. And based on safety data, the DSMB may recommend that the lead investigator modify or terminate the trial.

### Protocol amendments

During the course of the trial, any formal modifications to the protocol or informed consent form will be agreed by the research team, approved by the Ethics Committee to implementation, and reported to participants if necessary. It will also be updated in the Clinical Trials Registry.

## Discussion

Platelet activation and aggregation play an important role in atherosclerotic thrombosis. Therefore, antiplatelet is crucial to treat CHD and reduce the occurrence of ischemic events after PCI [13]. The study aims to investigate the safety and efficacy of rivaroxaban plus clopidogrel in Chinese CHD and GID patients undergoing PCI, and whether the same anti-ischemic effect as standard DAPT therapy is obtained without increasing the risk of GIB in patients with gastrointestinal injury. Conventional platelet oxidase inhibitors, such as aspirin, can damage the gastrointestinal mucosa and are 4–6 times more likely to cause gastrointestinal injury due to anti-platelet activation. While P2Y_12_ receptor antagonists, such as clopidogrel, also have an increased risk of GIB, but by a different mechanism from aspirin, increasing the risk of gastrointestinal injury secondary to impaired platelet-derived growth factor release. These growth factors promote endothelial cell proliferation and accelerate mucosal healing. Clopidogrel can make gastrointestinal injury more severe in combination with aspirin [14]. Previous studies have demonstrated the safety and efficacy of NOAC in combination with an antiplatelet agent in patients with CHD and atrial fibrillation (AF), but no studies have further analyzed the safety and efficacy of NOAC plus clopidogrel in CHD and GID. No increase or even reduces the risk of gastrointestinal bleeding in patients with GID and obtains the same anti-ischemic effect as conventional DAPT (Table 3). The anti-ischemic effect of DAPT, especially within 30 days, was investigated.

**Table 3:** Recent clinical studies on antithrombotic regimens in patients receiving long-term OAC therapy

Study title (year)	Patient population	Method	Conclusion
**WOEST (2013) **[9]	573 patients treated with OAC and undergoing PCI	① OAC combined with clopidogrel; ② OAC combined with clopidogrel and aspirin. Antiplatelet therapy in SCAD patients (1 month–1 year); at least 1 year of clopidogrel treatment in ACS patients	Dual antithrombotic reduces bleeding complications (19.4% VS. 44.4%, HR = 0.36, 95% CI 0.26 ~ 0.50, *P* < 0.0001), does not increase the incidence of thrombotic events
**PIONEER AF-PCI (2016) **[10]	2124 patients with PCI in combination with AF	① Rivaroxaban (15 mg qd) in combination with clopidogrel for 12 months; ② rivaroxaban (2.5 mg bid) in combination with DAPT for 1, 6, or, 12 months; ③ VKA combined with DAPT treatment for 1, 6, or 12 months	The incidence of clinically significant bleeding was lower in both rivaroxaban groups than in the standard triple antithrombotic group (16.8% vs. 18.0% vs. 26.7%; HR 0.59 and 0.63, respectively); central mortality was similar in the 3 groups
**RE-DUAL PCI (2017) **[15]	2725 patients with non-valvular AF who underwent PCI	① Dabigatran (100 mg bid) in combination with P2Y_12_ receptor inhibitor; ② dabigatran (150 mg bid) in combination with P2Y_12_ receptor inhibitor; ③ warfarin combined with DAPT	Dual antithrombotic is not inferior to triple antithrombotic in reducing the risk of ischemia, and the risk of bleeding is lower than in the triple antithrombotic group (15.4%VS. 26.9%, HR = 0.52, 95% CI 0.42 ~ 0.63, *P* < 0.0001)
**ENTRUST-AF PCI (2019) **[16]	1506 patients with AF who underwent PCI	① Edoxaban (60 mg qd) in combination with P2Y_12_ receptor inhibitor; ② VKA United DAPT. Aspirin therapy for 1 to 12 months (physician weighs), remaining medications for 12 months	Dual antithrombotic is non-inferior to triple antithrombotic in terms of bleeding (17% VS. 20%, *HR* = 0.83, 95% *CI* 0.65 ~ 1.05, non-inferior *P* = 0.001), no statistically significant difference in terms of ischemic events
**AUGUSTUS (2019) **[17]	4614 patients with AF with a recent history of ACS or PCI and treated with a P2Y_12_ receptor inhibitor (clopidogrel was chosen in 92.6% of patients)	Apixaban (5 mg or 2 mg bid depending on the patient) or VKA combined with aspirin or placebo (2 × 2 analysis design, 4 groups) for 6 months	P2Y_12_ receptor inhibitor-based apixaban is superior to dual antithrombotic with VKA and triple antithrombotic with aspirin
**Gemini-ACS-1 (2017) **[11]	3027 patients with ACS	① Received low-dose rivaroxaban (2.5 mg qb) plus a P2Y_12_ inhibitor (clopidogrel or tigretol); ② Aspirin (100 mg bid) plus P2Y_12_ inhibitor(clopidogrel or tigretol)	A dual pathway antithrombotic therapy approach combining low-dose rivaroxaban with a P2Y_12_ inhibitor for the treatment of patients with acute coronary syndromes had a similar risk of clinically significant bleeding as aspirin and a P2Y_12_ inhibitor

*OAC* oral anticoagulant, *PCI* percutaneous coronary intervention, *AF* atrial fibrillation, *ACS* acute coronary syndrome, *SCAD* stable coronary artery disease, *qd* once daily, *bid* twice daily, *DAPT* dual antiplatelet therapy, *VKA* vitamin K antagonist, *DES* drug-eluting stent

Bleeding events after PCI are independently associated with increased mortality, bleeding has become an important prognostic indicator for patients with ACS, and reducing bleeding events has become a major goal to improve the outcome of antiplatelet therapy. Bleeding events reduce patient compliance with medication and further increase prognostic risk. Although studies have confirmed the protective effect of PPI on the drug-induced injury of the gastrointestinal tract (such as aspirin and clopidogrel) [5], clopidogrel combined with PPI significantly reduces gastrointestinal adverse effects and has no significant differences in cardiovascular events. But pharmacokinetics studies have shown that some PPIs even can affect the antiplatelet reactivity of clopidogrel. Not all patients get the benefit from concomitant antiacid therapy [18]. Furthermore, PPI application does not fundamentally reduce the risk of bleeding associated with DAPT. It is noteworthy that anticoagulants do not cause gastrointestinal damage.

The 2020 ESC guidelines for atrial fibrillation recommend discontinuing aspirin within 1-week and continuing (N)OAC in combination with a P2Y_12_ receptor inhibitor for up to 12 months in patients with atrial fibrillation and ACS parallel uncomplicated PCI if the risk of stent thrombosis is low or the risk of bleeding is higher than the risk of ischemia [19]. Previous studies in patients with atrial fibrillation combined with CHD, including WOEST [9], PIONEER AF-PCI [10], RE-DUAL PCI [15], ENTRUST-AF PCI [16], AUGUSTUS [17], and GEMINI-ACS-1 [11], have demonstrated that OAC in combination with an antiplatelet agent is more effective than conventional DAPT or triple OAC + DAPT. But these studies were heterogeneous with the Asian yellow population, and most of the studies enrolled Caucasians [9–11, 15–17]. Rivaroxaban is the first oral, direct Factor Xa inhibitor to receive approval from regulatory authorities for stroke prevention in patients with AF, based mainly on the Phase III results of the ROCKET AF trial [20]. Rivaroxaban has certain advantages over warfarin and other VKAs in efficacy and safety in diseases at high risk of thrombosis, including ACS. Since factor Xa plays a central role in thrombosis, inhibition of factor Xa with low-dose rivaroxaban might improve cardiovascular outcomes in patients with recent ACS. It has the advantages of rapid onset of action, predictable efficacy, no need for routine coagulation monitoring, and conventional-dose adjustment [21, 22].

Dual antithrombotic therapy with rivaroxaban 15 mg once daily plus clopidogrel is a feasible treatment strategy among patients undergoing PCI with AF in the PIONEER AF-PCI trial [10]. The study of Lin et al. [23] demonstrated that low-dose rivaroxaban 10 mg once daily was associated with equal risks of ischemic stroke, systemic embolism, or major and nonmajor clinically relevant bleeding compared with the standard dose rivaroxaban 15 mg or 20 mg once daily in Asian patients with AF. There are a lower risk of ischemia and a higher risk of bleeding in Asian populations. In this study, patients undergoing PCI without AF are receiving clopidogrel and rivaroxaban 10 mg once daily in terms of efficacy and safety.

If the results of this study are reasonable, it will provide a new strategy for the secondary prevention of CHD combined with GID undergoing PCI and more evidence for guidelines.

## Conclusion

This study is the first trial to investigate the safety and efficacy of rivaroxaban plus clopidogrel as an alternative to DAPT after PCI in patients with CHD and GID.

## Trial status

This study is under participant recruitment. The first patient was included on 8th Apr. 2021, and the recruitment will be finished on 31st Dec. 2023. At present, 720 patients have been recruited, and 506 of them with complete follow-up visits of 6 months.

### Dissemination policy

The results of the study will be disseminated to participants, the public, and other relevant groups through publications and presented at scientific conferences.

## Supplementary Information

**Additional file 1.** SPIRIT checklist.

**Additional file 2.** Informed consent.

**Additional file 3.** Endpoint Definitions.

## Abbreviations

DAPT: Dual antiplatelet therapy
CHD: Coronary atherosclerotic heart disease
PCI: Percutaneous coronary intervention
GID: Gastrointestinal diseases
BARC: Bleeding Academic Research Consortium
MACCE: Major adverse cardiovascular and cerebrovascular events
UGIB: Upper gastrointestinal bleeding
ACS: Acute coronary syndrome
NSTE-ACS: Non-ST segment elevation acute coronary syndromes
GRACE: Global Registry of Acute Coronary Events
NSTEMI: Non-ST segment elevation myocardial infarction
STEMI: ST-segment elevation myocardial infarction
PPI: Proton pump inhibitors
ITT: Intention-to-treat
PP: Per-protocol
CEC: Clinical Events Committee
DSMB: Data and Safety Monitoring Boards
VKA: Vitamin K antagonist
OAC: Oral anticoagulant
NOAC: New oral anticoagulant
AF: Atrial fibrillation
