# Supplementary material for: Efficacy and safety of rivaroxaban plus clopidogrel versus aspirin plus clopidogrel in patients with coronary atherosclerotic heart disease and gastrointestinal disease undergoing percutaneous coronary intervention: study protocol for a non-inferiority randomized controlled trial
Source: Trials. 2023 Mar 21;24:209. doi: 10.1186/s13063-023-07236-w (PMC10031942; doi:10.1186/s13063-023-07236-w)
Supplement: Supplementary file 2 — Additional file 2. Informed consent. [file 13063_2023_7236_MOESM2_ESM.docx]

**Informed Consent**

| Project title | The efficacy and safety of clopidogrel combined with rivaroxaban and aspirin in patients with coronary heart disease undergoing PCI: a randomized controlled study |
| --- | --- |
| Version and Date | Version NO.V3.1  Version date 29th Jan.2021 |
| Sponsor | Ward 5, Department of Cardiology, General Hospital of Northern Theater Command |
| Clinical trial institution | The General Hospital of Northern Theater Command |
| Investigator |  |
| Screening number |  |

Dear __________ Sir/Madam:

You are invited to participate in the study, the efficacy and safety of clopidogrel combined with rivaroxaban and aspirin in patients with coronary heart disease undergoing PCI: a randomized controlled study, carried out by the General Hospital of the Northern Theater Command (former the General Hospital of Shenyang Military Region). Your condition meets the criteria of this study. The principal investigator of this study is __________ (phone number: _____________). The informed consent form will introduce the purpose, steps, benefits, risks, inconvenience, etc of the study to you. Please read it carefully before deciding whether to participate in the study. When medical workers explain and discuss the informed consent form with you, you can ask questions about what you don't understand. You can decide after discussing it with family, friends, and your attending doctor.

**Background**

In the past ten years, the occurrence of coronary atherosclerotic heart disease (CHD) has significantly increased in China, and the mortality rate is up to 15.2%. CHD remains the first cause of death in plenty of provinces and cities in our country. The basis of antithrombosis is aspirin combined with a P2Y_12_ inhibitor for patients with (CHD) undergoing percutaneous coronary intervention (PCI). It can reduce the risk of ischemic or atherothrombotic events (including stent thrombosis, recurrent myocardial infarction, and cardiac death). The Chinese guidelines for PCI (2016) recommend patients with stable coronary artery disease after undergoing PCI need to take dual antiplatelet therapy (DAPT) for 6 months. To prevent stent thrombosis events, patients with acute coronary syndrome after undergoing PCI require DAPT for 12 months. Aspirin can inhibit the activity of cyclooxygenase in the gastrointestinal tract, cause gastrointestinal mucosal epithelial damage, and then cause bleeding. Patients with gastrointestinal diseases (GID) undergoing PCI take DAPT increasing the risk of bleeding and severely causing death. The bleeding events of 48.7% occurred in the gastrointestinal tract of patients undergoing PCI. Reducing gastrointestinal bleeding is extremely important during the perioperative period. In the past, patients with CHD who combined GID or could not tolerate aspirin were often treated with delayed PCI or conservative therapy. Previous studies show that comparing with the triple antithrombotic regimen, rivaroxaban and clopidogrel treatment have lower bleeding rate and the efficacy was not inferior in patients with CHD and atrial fibrillation undergoing PCI. Whether rivaroxaban can instead of aspirin in antithrombotic therapy for patients with CHD and GID, there is no research evidence and no relevant guideline.

**Objective**

The objective of this study is to evaluate that comparing with aspirin plus clopidogrel, the efficacy and safety of rivaroxaban plus clopidogrel in patients with CHD and GID undergoing PCI.

**Method and Content**

A total of 1020 patients with CHD and GID undergoing PCI will be enrolled in this study. The study is a prospective, randomized controlled trial. The patients meet all inclusion criteria and no exclusion criteria are eligible for this study after signing the informed consent form. Patients are randomized (1:1) to the experiment arm (rivaroxaban 10mg plus clopidogrel 75mg daily) or the control arm (aspirin 100mg plus clopidogrel 75mg daily).

**Proceeding and Deadline**

Treatment of the subject will last 6 months. At the end of the study, the attending doctor decides to follow antithrombotic treatment of the subject. After randomization and before treating with the medical of the study (visit 1), investigators will collect the general clinical information and information of PCI. At the follow-up visit of 30 days by telephone contact (visit 2), investigators will know about the subjects whether occur bleeding events, or cardiovascular and cerebrovascular adverse events (MACCE). And investigators should record the details including the location, time, signs, duration, progress, etc. At the follow-up visit of 6 months on-site (visit 3), subjects need to accept routine blood and fecal occult blood examination. investigators will know about and record content the same as visit 2.

**Possible Benefits**

If you participate in this study, you will get standard follow-up from cardiologists and nurses. It may reduce the occurrence of cardiovascular and cerebrovascular adverse events, and improve the quality of life. On account of your participation, investigators may have a deep understanding of antithrombotic treatment and further reduce the occurrence of adverse events for patients with CHD and GID undergoing PCI.

**Possible Risks**

This study aims to evaluate the safety and efficacy of rivaroxaban plus clopidogrel and aspirin plus clopidogrel in patients with CAD and GID undergoing PCI. It hardly impact on your life. You may feel fatigued during the questionnaire and study. If you feel severely fatigued or uncomfortable, please immediately inform medical workers. And if you have any questions about the checks and progress of this study, you can consult us anytime.

**Other Risks**

Some questions in the questionnaire may make you uncomfortable and you can refuse to answer them.

**Alternative Therapy**

It will not have any effect on the standard treatment that you choose don’t participate in this study. Your attending doctor will determine your antithrombotic treatment.

**Remuneration or Additional cost**

You will not get paid for participating in this study. The cost of your surgery and daily care will be charged according to the hospital's routine. You will get the free drug clopidogrel of this study for a week. After 6 months of treatment, you will need to return to the hospital and run a blood routine and fecal occult blood test.

**Access and Confidentiality of medical records**

Your personal information will be kept confidential in this study. When recording study results, the study center code and your initials will be used to represent your personal information. The sponsor, ethics committees, clinical drug trial institutions, State Food and Drug Administration, and drug administration departments may access your original medical records for verifying the information collected or managing the clinical trial.

If you agree to participate in this study and sign the informed consent form, the sponsor and other above-mentioned personnel can view and copy your medical records including your preoperative and postoperative physical conditions, follow-up examinations, and hospital bills. For the purpose of the academy, the Research Committee may review partial medical records (such as discharge reports or electrocardiograms, etc.) that contain your name. All patient data will be kept strictly confidential in accordance with local data protection laws. There is no time limit to the use of health information.

All medical data about you will be kept by the sponsor in secure computer files that will be analyzed along with data from other participants. Your name and any other personal data will not appear in these files. The results of this study may be published. At that time your identity will not be revealed.

**Volunteering or Withdrawing**

You are free to participate in this study. Refusing participation will not have any negative impact on your current or future medical care. If you agree to take part, you can change your mind and tell medical workers to quit the study at any time. It also don’t affect your standard of care.

After knowing the details of the study, you have the right of refusing to sign the informed consent. If you have any questions about this study, please contact medical workers. Contact as below:

Investigator: Phone number:

If you have questions about the rights of the subjects, you can contact the Ethics Committee of General Hospital of Northern Theater Command.

Phone number:

**Informed Consent Signing Page**

**Investigator Statement**

“I have informed the subject the following information: (1) The background, purpose, risks, and benefits of the study; (2) The subject has sufficient time to read the informed consent, discuss it with others, and ask questions about the study; (3) The subject can contact doctors and nurses at any time when encountering problems related to the project, and contact the Ethics Committee of General Hospital of Northern Theater Command at any time when encountering problems related to their rights. (4) Relevant contact information; (5) The subject is free to withdraw from the study; (6) The subject will get a copy signed by researcher and him/her of this informed consent.

Signature of investigator: Data:

**Subject Statement**

“I have been informed of the following information: (1) The background, purpose, risks, and benefits of the study; (2) Having sufficient time and opportunity to ask questions, and getting satisfactory answers; (3) Relevant contact information; (4) I can withdraw from the study at any time without any reason; (5) I will get a copy which signed by me and the investigator of this informed consent. I have read this informed consent and agree to participate in this study.

Signature of the subject: Data:

Phone number:

**(When informed consent ability of subject is insufficient, adding or replacing the following method)**

Signature of family or authorized representative:

Data: Phone number:

Relationship with the subject:

**知情同意书**

| **方案名称** | 氯吡格雷联合利伐沙班与联合阿司匹林在冠心病伴有胃肠疾病患者行PCI治疗的有效性及安全性研究：一项随机对照研究 |
| --- | --- |
| **知情同意书版本及日期：** | 版本号V3.1 版本日期 2021.01.29 |
| **申办者** | 北部战区医院心内科五病区 |
| **临床试验机构** | 北部战区总医院 |
| **研究者** |  |
| **筛选号** |  |

尊敬的 先生/女士：

您被邀请参与由北部战区总医院（原沈阳军区总医院）开展的氯吡格雷联合利伐沙班与联合阿司匹林在冠心病伴有胃肠疾病患者行PCI治疗的有效性及安全性研究治疗项目，您的具体情况符合该项项目的入组条件，本项目负责人为__________（联系电话：______________）。本知情同意书将向您介绍该项目的目的、步骤、获益、风险、不便或不适等，请仔细阅读后慎重做出是否参加项目的决定。当医护人员向您说明和讨论知情同意书时，您可以随时提问并让他/她向您解释您不明白的地方。您可以与家人、朋友以及您的经治大夫讨论之后再做决定。

**项目背景：**

冠心病在我国近十年来发病率明显升高，死亡率高达15.2%，是我国多个省市的首位死亡原因。阿司匹林联合P2Y12受体拮抗剂是冠心病行经皮冠状动脉介入治疗（percutaneous coronary intervention, PCI）的抗栓基础，可降低缺血性或动脉粥样硬化血栓事件的风险，包括支架内血栓形成、复发性心肌梗死及心源性死亡。中国经皮冠状动脉介入治疗指南(2016)推荐稳定性冠心病行PCI治疗需双联抗血小板6个月。急性冠脉综合征患者行PCI治疗需双联抗血小板12个月，预防支架内血栓事件发生。因阿司匹林能抑制胃肠道环氧合酶的活性，引起胃肠粘膜上皮损伤致出血，使得双联抗血小板(dual antiplatelet therapy，DAPT)增加PCI后伴有胃肠疾病的患者出血的风险，严重者导致死亡。PCI患者出血事件中发生在消化道占48.7%，围术期降低消化道出血极为重要。以往在临床上伴有胃肠疾病及不能耐受阿司匹林的冠心病患者往往采用延迟行PCI或药物保守治疗，预后极差。在冠心病合并心房颤动行PCI治疗的患者中利伐沙班联合氯吡格雷的研究中得出的出血发生率低、疗效不劣于三联抗栓方案的结论。对于冠心病伴有胃肠疾病是否能应用利伐沙班替代阿司匹林，但目前无研究证据，无相关指南推荐。

**项目目的：**

在中国北部战区总医院（原沈阳军区总医院）开展氯吡格雷联合利伐沙班与联合阿司匹林在冠心病伴有胃肠疾病患者行PCI治疗的有效性及安全性研究项目，本研究的目的是：与氯吡格雷联合阿司匹林相比，评价氯吡格雷联合利伐沙班在中国伴有胃肠疾病的行经皮冠状动脉介入治疗（percutaneous coronary intervention, PCI）的冠心病患者中应用的安全性和有效性。

**试验****方法与内容**

本项目计划招募1020名受试者。

本项目采用前瞻性、随机对照设计，患者签署知情同意书后，符合临床入选标准但不符合临床排除标准的患者（1020例）在入院接受PCI治疗后，按照1：1的比例，将其随机分为两组：试验组，氯吡格雷联合利伐沙班治疗组（510例）接受氯吡格雷 75mg 1/日和利伐沙班10mg 1/日研究用药物的治疗和对照组，氯吡格雷联合阿司匹林治疗组（510例）接受氯吡格雷 75mg 1/日和阿司匹林 100mg 1/日研究用药物治疗。

**项目****过程和期限**

患者按要求每日用药直至研究结束。6个月以后的抗栓治疗由患者主治医生自行决定。在随机化分组后未进行试验时，要进行一次访视（访视1），主要收集患者的一般临床资料、PCI相关资料。在随机化分组治疗药物服用后的30天以及1个月对所有患者进行一次电话或住院访视（访视2、访视3、访视4），收集患者对应时间点发生的出血事件、心脑血管不良事件情况，并进行记录。记录内容包括：发生部位、时间、体征、持续天数、是否好转、是否就医等问题。随机分组治疗后6月：患者需返回医院，进行血常规以及便潜血检查，同时询问患者是否发生的出血事件、心脑血管不良事件情况，如有发生，询问其发生部位、时间、体征、持续天数、是否好转、是否就医等问题。

**可能的受益**

参与本项目，您将得到心内科专科医生、护士的正规随访，这可能有助于减少心脑血管不良事件发生率和提高生活质量。由于您的参与可能会增加研究者对于这类合并有胃肠疾病的冠心病患者PCI术后抗栓药物选择的了解，使这一人群获益，减少不良事件的发生。

**可能的风险**

本项目旨在评价氯吡格雷联合利伐沙班与联合阿司匹林在冠心病伴有胃肠疾病患者行PCI治疗的有效性及安全性研究的安全性和有效性 ，几乎不会对您的生活产生任何影响。您可能在问卷调查和学习期间感到有些疲劳，如果疲劳感严重或有其他不适症状，请立刻告知医护人员。若您对试验涉及的检查和步骤有任何疑问，可以向我们咨询。

**其他风险：**

问卷中的某些问题可能会让您感到不舒服，您可以拒绝回答。

**替代疗法**

您可以选择不参加本项目，这对您获得常规治疗不会带来任何不良影响。如您不参与本项目，将由您的主治医生决定您的抗栓治疗方案。

**项目期间获得****报酬或额外费用**

您参与这项项目不会获得报酬，无论您是否参与本研究，您的手术费用和日常护理的费用将按医院常规收取，手术和护理费用将由您自行承担。但您可获得试验用药氯吡格雷免费一周。您需要在治疗6个月后返回医院，进行血常规以及便潜血检查。

**医疗记录的获取和保密性**

本研究将为收集到的关于您的个人信息进行保密。在记录研究结果时，将仅使用研究中心代码和您的姓名缩写来代表您的个人信息，申办方或其代表、伦理委员会、临床药物试验机构、国家食品药品监督管理局及药政部门为核实收集的信息或出于临床试验管理的需要可能查阅您的原始医疗记录。

通过参与本研究及本知情同意书，表示您同意并签字确认允许申办方等上述人员查阅和复制您的医疗记录，包括您术前术后的身体状况、随访检查和化验、住院医疗账单。研究委员会出于学术目的可能会审查不含有您名字的相关的部分医疗记录复印件（如出院报告或心电图等）。所有患者的资料都将根据当地的数据保护法律严格保密。健康信息的使用没有期限。

所有有关您的医疗数据将由申办单位保存在安全的计算机文件中，将与其他参与者的数据一同进行分析，但您的名字和任何其它可能识别您身份的个人数据不会出现在这些文件中。本研究的结果可能会发表，但您的身份不会被暴露。这些数据可能会提供给国家食品药品管理总局用于该产品在国内注册，经审批后以备将来上市。

**自愿参加或退出项目**

您是否参加这个项目完全是自愿的。如果您不愿意，可以拒绝参加，这对您目前或未来的医疗不会有任何负面影响。即使您同意参加之后，您也可以在任何时间改变主意，告诉医护人员退出项目，这同样不会影响您获得正常的医疗服务。

在了解项目的详细信息后，您有权拒绝签署同意书。如果您有与本项目相关的任何问题，请联系医护人员。联系方式如下：

研究者： 联系电话：

如果您有与受试者自身权益相关的问题，可与北部战区总医院临床项目伦理委员会联，联系电话：

**知情同意书签署页**

# 研究者声明

“我已告知氯吡格雷联合利伐沙班与联合阿司匹林在冠心病伴有胃肠疾病患者行PCI治疗的有效性及安全性研究项目背景、目的、风险及获益情况，给予他/她足够的时间阅读知情同意书、与他人讨论，并解答了其有关项目的问题；我已告知该受试者当遇到与项目相关的问题时可随时与医生、护士联系，遇到与自身权利/权益相关问题时随时与北部战区总医院医院伦理委员会联系，并提供了准确的联系方式；我已告知该受试者他/她可以退出本项目；我已告知该受试者他/她将得到这份知情同意书的副本，上面包含我和他/她的签名。”

项目者签名： 日期

# 受试者声明

“我已被告知氯吡格雷联合利伐沙班与联合阿司匹林在冠心病伴有胃肠疾病患者行PCI治疗的有效性及安全性研究项目背景、目的、步骤、风险及获益情况。我有足够的时间和机会进行提问，问题的答复我很满意。我也被告知，当我有问题、想反映困难、顾虑、对项目的建议，或想进一步获得信息，或为项目提供帮助时，应当与谁联系。我已经阅读这份知情同意书，并且同意参加本项目。我知道我可以在项目期间的任何时候无需任何理由退出本项目。我被告知我将得到这份知情同意书的副本，上面包含我和项目者的签名。”

受试者签名： 签名日期：

联系电话：

**（当受试者知情同意能力欠缺或不足时，增加或替换以下方式：）**

家属或授权代表签名： 签名日期：

联系电话：

与受试者关系：
