# Supplementary material for: Efficacy and safety of rivaroxaban plus clopidogrel versus aspirin plus clopidogrel in patients with coronary atherosclerotic heart disease and gastrointestinal disease undergoing percutaneous coronary intervention: study protocol for a non-inferiority randomized controlled trial
Source: Trials. 2023 Mar 21;24:209. doi: 10.1186/s13063-023-07236-w (PMC10031942; doi:10.1186/s13063-023-07236-w)
Supplement: Supplementary file 3 — Additional file 3. Endpoint Definitions. [file 13063_2023_7236_MOESM3_ESM.docx]

**Endpoint Definitions**

1. **Bleeding according to Bleeding Academic Research Consortium Criteria^[1]^**

**Type 0:** No bleeding.

**Type 1:** Bleeding that is not actionable and does not cause the patient to seek unscheduled performance of studies, hospitalization, or treatment by a health care professional; may include episodes leading to self-discontinuation of medical therapy by the patient without consulting a health care professional.

**Type 2:** Overt, actionable sign of hemorrhage (e.g., more bleeding than would be expected for a clinical circumstance, including bleeding found by imaging alone) that does not fit the criteria for type 3, 4, or 5 but does meet at least one of the following criteria: (1) requiring nonsurgical, medical intervention by a health care professional, (2) leading to hospitalization or increased level of care, or (3) prompting evaluation.

**Type 3:**

Type 3a: Overt bleeding plus hemoglobin drop of 3 to < 5 g/dL (provided hemoglobin drop is related to bleed). Any transfusion with overt bleeding.

Type 3b: Overt bleeding plus hemoglobin drop ≥5 g/dL (provided hemoglobin drop is related to bleed), cardiac tamponade, bleeding requiring surgical intervention for control (excluding dental/nasal/skin/hemorrhoid), bleeding requiring intravenous vasoactive agents.

Type 3c: Intracranial hemorrhage (does not include microbleeds or hemorrhagic transformation, does include intraspinal). Subcategories confirmed by autopsy or imaging or lumbar puncture intraocular bleed compromising vision.

**Type 4:** CABG-related bleeding:

- Perioperative intracranial bleeding within 48 hours.

- Reoperation after the closure of sternotomy to control bleeding.

- Transfusion of ≥5 U whole blood or packed red blood cells within a 48 hour period.

- Chest tube output ≥2 L within 24 hour-period.

**Type 5:** Fatal bleeding.

Type 5a: Probable fatal bleeding; no autopsy or imaging confirmation but clinically suspicious.

Type 5b: Defifinite fatal bleeding; overt bleeding or autopsy or imaging confirmation.

1. **MACCE**
2. **Death^[2]^**

Classifications of death:

**Cardiac death:**

Any death due to proximate cardiac cause (e.g., myocardial infarction, low-output failure, fatal arrhythmia), unwitnessed death, and death of unknown cause, and all procedure-related deaths, including those related to concomitant treatment, will be classified as cardiac death.

**Vascular death:**

Death is caused by noncoronary vascular causes, such as cerebrovascular disease, pulmonary embolism, ruptured aortic aneurysm, dissecting aneurysm, or other vascular diseases.

**Non-cardiovascular death:**

Any death not covered by the above definitions, such as death caused by infection, malignancy, sepsis, pulmonary causes, accident, suicide, or trauma.

All deaths are considered cardiac unless an unequivocal noncardiac cause can be established. Specifically, any unexpected death even in patients with coexisting potentially fatal noncardiac disease (e.g., cancer, infection) is classified as cardiac.

1. **Myocardial Infarction^[3]^**

Based on the third universal definition, myocardial infarction will be classified into various types:

**Type 1:** Spontaneous myocardial infarction

Spontaneous myocardial infarction related to atherosclerotic plaque rupture, ulceration, fissuring, erosion, or dissection with resulting intraluminal thrombus in one or more of the coronary arteries leading to decreased myocardial blood flow or distal platelet emboli with ensuing myocyte necrosis.

The patient may have underlying severe CAD but on occasion nonobstructive or no CAD.

**Type 2:** Myocardial infarction secondary to an ischemic imbalance

In instances of myocardial injury with necrosis where a condition other than CAD contributes to an imbalance between myocardial oxygen supply and/or demand, e.g., coronary endothelial dysfunction, coronary artery spasm, coronary embolism, tachy-/bradyarrhythmias, anemia, respiratory failure, hypotension, and hypertension with or without left ventricular hypertrophy (LVH).

**Type 3:** Myocardial infarction resulting in death when biomarker values are unavailable

Cardiac death with symptoms suggestive of myocardial ischemia and presumed new ischemic ECG changes or new LBBB, but death occurring before blood samples could be obtained, before cardiac biomarkers could rise, or in rare cases cardiac biomarkers were not collected.

**Type 4a:** Myocardial infarction related to the percutaneous coronary intervention (PCI)

Myocardial infarction associated with PCI is arbitrarily defined by the elevation of cTn values > 5 × 99th percentile URL in patients with normal baseline values (< 99th percentile URL) or a rise of cTn values > 20% if the baseline values are elevated and are stable or falling. In addition, either (1) symptoms suggestive of myocardial ischemia, (2) new ischemic ECG changes or new LBBB, or (3) angiographic loss of patency of a major coronary artery or a side branch or persistent slow- or no-flow or embolization, or (4) imaging demonstration of new loss of viable myocardium or new regional wall motion abnormality are required.

**Type 4b:** Myocardial infarction related to stent thrombosis

Myocardial infarction associated with stent thrombosis is detected by coronary angiography or autopsy in the setting of myocardial ischemia and with a rise and/or fall of cardiac biomarkers values with at least one value above the 99th percentile URL.

**Type 5:** Myocardial infarction related to coronary artery bypass grafting (CABG)

Myocardial infarction associated with CABG is arbitrarily defined by the elevation of cardiac biomarker values > 10× 99 the percentile URL in patients with normal baseline cTn values (< 99th percentile URL). In addition, either (1) new pathological Q waves or new LBBB, or (2) angiographic documented new graft or new native coronary artery occlusion, or (3) imaging evidence of new loss of viable myocardium or new regional wall motion abnormality.

Q-wave versus non-Q-wave myocardial infarction

Q-wave MI will be diagnosed if new pathologic Q-waves (≥25% of the height of the partner R wave and/or ≥0.04 seconds in duration) in ≥2 contiguous ECG leads occur. All other MIs not fulfilling the above-mentioned criteria will be considered non-Q-wave MI.

Criteria for acute myocardial infarction include:

Detection of a rise and/or fall of cardiac biomarker values (preferably cardiac troponin [cTn]) with at least one value above the 99th percentile upper reference limit (URL) and with at least one of the following:

- Symptoms of ischemia.

- New or presumed new significant ST-segment–T wave (ST–T) changes or new left bundle branch block (LBBB).

- Development of pathological Q waves in the electrocardiogram (ECG).

- Imaging evidence of new loss of viable myocardium or new regional wall motion abnormality.

- Identification of an intracoronary thrombus by angiography or autopsy.

- Cardiac death with symptoms suggestive of myocardial ischemia and presumed new ischemic ECG changes or new LBBB, but death occurred before cardiac biomarkers were obtained, or before cardiac biomarker values would be increased.

1. **Stroke^[4]^**

A stroke is defined as an acute episode of focal or global neurological dysfunction of at least 24 hours caused by the brain, spinal cord, or retinal vascular injury as a result of hemorrhage or infarction.

1. **Stent Thrombosis^[2]^**

According to Academic Research Consortium (ARC):

- Definite stent thrombosis: Presence of an acute coronary syndrome with angiographic or autopsy evidence of thrombus or occlusion.

- Probable stent thrombosis: Unexplained death within 30 days after the procedure or acute myocardial infarction involving the target-vessel territory without angiographic confirmation.

- Possible stent thrombosis: All unexplained death occur within 30 days after the procedure.

- Acute stent thrombosis: Occurring within 24 hours following the index PCI.

- Subacute stent thrombosis: 24 hours to 30 days following the index PCI.

- Late stent thrombosis: 31 to 360 days following the index PCI.

- Very late stent thrombosis: after 360 days following the index PCI.

1. **Ischemia-driven Target Vessel Revascularization**

Ischemia-driven target vessel revascularization is defined as repeat PCI or bypass surgery of the target lesion(s) and any additional lesions in the main epicardial coronary artery or branches containing the target lesion.

**References**

[1]Mehran R, Rao SV, Bhatt DL, et al. Standardized bleeding definitions for cardiovascular clinical trials: a consensus report from the Bleeding Academic Research Consortium. Circulation 2011;123 (23):2736–2747

[2]Cutlip DE, Windecker S, Mehran R, et al; Academic Research Consortium. Clinical endpoints in coronary stent trials: a case for standardized definitions. Circulation 2007;115(17):2344–2351

[3]Thygesen K, Alpert JS, Jaffe AS, et al; Joint ESC/ACCF/AHA/WHF Task Force for Universal Definition of Myocardial Infarction; Authors/Task Force Members Chairpersons; Biomarker Subcom-mittee; ECG Subcommittee; Imaging Subcommittee; Classification Subcommittee; Intervention Subcommittee; Trials & Registries Subcommittee; Trials & Registries Subcommittee; Trials & Registries Subcommittee; Trials & Registries Subcommittee; ESC Committee for Practice Guidelines (CPG); Document Reviewers. Third universal definition of myocardial infarction. J Am Coll Cardiol 2012;60(16):1581–1598

[4]Hicks KA, Mahaffey KW, Mehran R, et al; Standardized Data Collection for Cardiovascular Trials Initiative (SCTI). 2017 Cardiovascular and Stroke Endpoint definitions for clinical trials. Circulation 2018;137(09):961–972
